# Identity as a resource or a demand

**DOI:** 10.1371/journal.pone.0318449

**Published:** 2025-01-28

**Authors:** Karishma K. Singh, Andrew J. Elliot, Elizabeth D. Handley, Jeremy P. Jamieson

**Affiliations:** Department of Psychology, University of Rochester, Rochester, New York, United States of America; The Open University of Israel, ISRAEL

## Abstract

Individuals embody various social identities that can impact how they interface with the social environment. Stigma theories suggest that members of low-status or marginalized groups possess devalued social identities, and therefore, experience more stress. While social identities can lead to increased stress, individuals’ appraisals of their identities are not necessarily perceived as harmful/demanding. Rather, social identities can also be appraised as resources or sources of strength bringing opportunities and facilitating goal attainment. Using the biopsychosocial (BPS) model of challenge and threat as a conceptual foundation, this research developed a novel measure to assess individuals’ appraisals of their social identities. In Study 1 (N = 575), confirmatory factor analysis (CFA) evaluated the theorized factor structure (i.e., resource and demand appraisals) and assessed the overall fit of the model. Structural equation modeling (SEM) tested for associations between the resource and demand latent factors. Individuals appraising their ethnic-racial identity as a resource exhibited improved self and intergroup outcomes, while those perceiving it as a demand reported worse self-based and intergroup outcomes, as well as more distress. Study 2 (N = 743 Black and White Americans), which was preregistered, examined group differences in appraisals of ethnic-racial identity. SEM revealed that Black participants were more likely than White participants to appraise their ethnic-racial identity as demanding, leading to worse social and intergroup outcomes. Even when Black participants perceived their ethnic-racial identity as a resource, they still reported higher levels of discrimination, intergroup anxiety, and behavioral avoidance compared to their White counterparts. Implications for theory development and application to the stress literature are discussed.

## Introduction

Stress is ubiquitous in daily life. We routinely encounter situations that require us to marshal resources to address acute demands, such as interviewing for a new job, completing assignments at school or work, or resolving interpersonal conflicts. A large corpus of research has elucidated the negative cumulative health effects of encountering stressors (e.g., [1]), the cognitive processes that shape stress responses (e.g., [2]), and the myriad effects of acute stress responses on behavior, emotions, and decisions (e.g., [3]). However, a crucial and underexplored aspect of stress research is how social group identity processes determine how *individuals* respond to the stressors they face. This area of research is notable given the many and varied social identities that people hold, and the importance of how those social identities interface with stressful experiences (e.g., [4–6]). Understanding how individuals appraise their social identities, as sources of strength or as burdens, can have important implications for how they cope with stress.

To date, however, research has yet to examine how individuals’ appraisals of their social identities as coping resources *and* demands that must be overcome shape stress responses. This absence is significant because social identities are central to individuals self-concept, and understanding how people view their identity, whether as a resource or a demand, can offer new insights into how stress is experienced. That is, the extant research literature on social identity and stress lacks the perspective of the individual appraiser.

Using the Biopsychosocial (BPS) model of challenge and threat as an organizing framework, the research presented here investigated how individual appraisals interface with stress and intergroup outcomes. For example, consider appraisals of ethnic-racial identity. One’s ethnic-racial identity may be appraised as a valuable resource that provides social support, unique perspectives, and privileges, but one’s ethnic-racial identity may also be appraised as adding demands due to increased evaluative pressure, uncertainty about belonging, or stigmatization. Notably, these appraisals can be independent: an individual may simultaneously appraise their identity as a resource *and* a demand. Moreover, as emphasized in the BPS literature, appraisals are necessarily context-bound and variable [2]. The same identity that was appraised as more of a resource than demand in one context (e.g. a supportive social setting) may be appraised as more demanding in another (e.g. a competitive setting). Appraisals of our social identities can thus have far-reaching implications for social relationships and overall health.

Although stress is a central concept in this study, particularly through the lens of identity within stress appraisals, physiological stress indicators are not included. As a first step, this research focuses on measuring individual appraisals of identity, which lays the groundwork for future investigations into their physiological correlates. This research is the first to investigate how individual-level appraisals of social identities as resources or demands relates to experienced affect, well-being, and intergroup outcomes.

### Social identity processes

An individual’s self-concept derived from the social groups they belong to is known as their social identity [7]. Groups are characterized by members who share similar thoughts, behaviors, and perceptions [8]. Indeed, categorization of people into social groups is one of the most fundamental ways people understand each other, and people generate an internalized sense of membership to the social groups they belong to [9].

Researchers have argued that social groups can function as “social cures” providing individuals with a sense of purpose and belonging, enriching them, and making them stronger and healthier [10–12]. Therefore, having a positive view of the social groups one belongs to can positively impact overall health and well-being, and act as a psychological resource [13, 14]. Enhancement of self-esteem, mental health, and life efficacy can occur when individuals are part of groups they perceive as “superior to” or “better than” other groups [15–19]. Additionally, higher social status derived from social identities is connected to power and control over resources, as well as positive health trajectories [20–22].

Alternatively, while some identities can be advantageous, others have the potential to hinder growth or make one vulnerable to negative outcomes [23–25]. For instance, being a member of a disadvantaged or stigmatized social group can expose individuals to negative life circumstances and experiences which can threaten health and well-being [17, 19, 26]. Research on social identity threat suggests that when individuals are targeted by negative stereotypes, such as those related to math ability for women, it can lead to reduced motivation, lower feelings of belonging, and increased stress, all of which contribute to social withdrawal and diminished quality of life [27]. Therefore, group membership and identifying with stigmatized groups can also act as a “social curse,” adding stress and reducing self-esteem [28–30]. Similarly, the minority stress literature suggests stressful environments are directly created through experiences of stigma and discrimination that elicit negative mental health outcomes [31].

### BPS model of challenge and threat

Cognitive appraisal processes are central to understanding how people interface with their social worlds. Notably, biological stress systems that respond to environmental stressors do not have “eyes and ears”, but rather stress responses are shaped by appraisals of whether one can (i.e. approach) or cannot (i.e. avoid) address the stressors presented to them [2]. This distinction is crucial because stress responses are not merely automatic physiological reactions; they are influenced by how we interpret and assess the situations we encounter. BPS models—the dominant stress models in modern medicine—emphasize the importance of appraisals in stress responding. Indeed, the BPS model of challenge and threat has been used to understand a host of social stress responses, including but not limited to, competition, discrimination, and evaluative threat [32–34]. The BPS model of challenge and threat provides a foundational framework for understanding how appraising social identities as sources of resources and/or demands shapes how people respond to the stressors they encounter [35, 36].

Central to the BPS model of challenge and threat is the idea that appraisals of resources and demands are multifaceted, and can be both independent and intertwined [2]. For instance, resource appraisals include perceptions of skills or abilities, social support available, familiarity, etc., while demands encompass perceived effort, uncertainty, threats to social status, etc. Note that while skills/abilities are independent of social status threats, appraisals of familiarity and uncertainty are frequently related. Demand and resource appraisals then interact to determine stress responses. When resource appraisals exceed demand appraisals, individuals experience approach-oriented challenge-type responses, while avoidance-oriented threat responses manifest when resources are appraised as being insufficient to address demands [37]. Here, we posit that appraisals of social identities are part of the resource/demand calculus, and thus play a pivotal role in shaping individuals’ stress responses in contexts where social identities are salient and active.

The importance of understanding the breadth of demand and resource appraisals is vital because challenge-type stress responses predict a host of positive and health-protective processes and outcomes [37]. For instance, challenge leads to more efficient cardiac cycles (increased output, reduced vascular resistance) designed to deliver oxygenated blood to the brain and peripheral sites [38], as well as elevated levels of anabolic hormones relative to catabolic hormones [39]. Downstream benefits of challenge states extend beyond physiological efficiency; they also include improved cognitive performance, more positive emotional experiences, and a rapid return to homeostasis after stress offset [38, 40, 41]. Alternatively, experiences of threat that result when demands are perceived as exceeding resources elicit physiological responses aimed at protecting the individual from damage or social defeat, including high levels of vascular resistance to center blood in the core of the body and production of catabolic hormones such as cortisol [42]. Such threat responses can lead individuals to be hypervigilant for emotionally negative stimuli, experience reduced cognitive flexibility and remain in a state of heightened stress long after the stressor has been resolved (e.g., [3, 43, 44]).

While the interaction of resources and demands produce stress responses in BPS models and can be intertwined, it is important to consider these appraisals independently. Resources can increase independent of demands, and vice versa, highlighting the complexity of how individuals respond to different stressors. For instance, consider a student preparing for an exam. The student can devote more time towards studying and preparation to grow their knowledge (i.e., increase their resources), but this has no impact on the difficulty of the exam questions (a component of demands). Or, more relevant to the current research, the same social identity can be perceived as both a resource *and* a demand. For instance, while one may appraise their racial identity as a source of social support (i.e., a resource), they could also appraise their racial identity as causing demands associated with being a target of prejudice and discrimination. This duality of appraising one’s social identity as both a resource and a demand may explain how stress responses vary depending on context. If our understanding of resource and demand appraisals can be expanded and more precisely articulated, there is greater potential for intervening on these direct mechanisms to help people optimize their responses to stressors and regulate negative affective experiences [45].

### Current research

This research was designed with three central aims. First, given the absence of available measures to assess BPS-derived social identity appraisals, we developed an assessment to tap into individuals’ appraisals of their social identities as resources and demands. Second, we examined the association between social identity appraisals (ethnic-racial identity in particular) and affective, health, and intergroup processes. Finally, we examined an important source of heterogeneity in appraisals of ethnic-racial identity: participants’ membership in different racial/ethnic groups. To do so, we recruited large samples of Black and White Americans to examine whether resource and demand appraisals function similarly or differently across ethnic-racial identity, and how associations between resource and demand appraisals and psychological outcomes vary as a function of identity. This first test of the novel measure focused on ethnic-racial identity because this particular social identity is at the forefront of current American culture [46] and it is an “observable” identity, unlike other hidden identities such as sexual orientation [47]. Note that we focused on a U.S. sample because of the social and racial dynamics that influence identity appraisals and related outcomes in this context. The decision to sample Black and White participants in the U.S. was based on the aim to examine how these dynamics function in a society where race plays a significant role in shaping social experiences, particularly for racialized minorities. Additionally, the sample was not confined to a region of the United States but was drawn from people nationwide.

This research investigates how individual-level identity appraisals impact key intergroup outcomes: perceived discrimination, intergroup anxiety, intergroup mistrust, and behavioral avoidance. Perceived discrimination reflects individuals’ experiences of biased treatment in their environment [48]. Intergroup anxiety captures the apprehension that can hinder interactions [49, 50]. Intergroup mistrust can undermine trust between groups, reinforcing division and stereotypes [51, 52]. Lastly, behavioral avoidance reflects distancing from outgroup members [53, 54].

In addition to these focal outcomes, we examined subjective distress. Subjective distress captures an individual’s overall emotional burden, reflecting how these identity-based stressors impact broader, negative life stress perceptions [55]. Furthermore, we measured grit, defined as perseverance and passion for long-term goals, to examine how identity appraisals—viewing identity as a resource versus a demand—may influence an individual’s level of grit and resilience in the face of stress [56]. We also explored how identity appraisals influence both individual and collective self-esteem, as evaluations of the self can significantly affect these outcomes [57, 58].

We hypothesize that individuals who perceive their ethnic-racial identities as more of a resource will exhibit more positive psychological outcomes, including higher collective self-esteem, as well as lower discrimination, intergroup anxiety, interracial mistrust, and behavioral avoidance. In contrast, individuals who perceive their ethnic-racial identities as more demanding will exhibit worse social outcomes, such as higher discrimination, intergroup anxiety, interracial mistrust and behavioral avoidance, as well as lower collective self-esteem. Furthermore, we hypothesize that individuals holding stigmatized ethnic-racial identities (Black Americans) will perceive their racial identity as more demanding compared to a sample of non-Hispanic White Americans. Regarding moderation by ethnic-racial identity, White individuals who perceive their identity as more resourceful were hypothesized to report better psychological outcomes than Black individuals who perceived their identity as resourceful. Similarly, Black individuals who perceive their identity as more demanding were hypothesized to report worse psychological outcomes than White individuals who perceive their identity as demanding.

## Study 1

Study 1 tested the scale’s reliability and validity.

### Method

#### Preregistration and open science

All data for this and the following study were collected before any analyses were conducted. All data exclusions and variables analyzed are reported, and analyses were planned *a priori* with some deviations that are addressed throughout the paper. Study 1 recruitment period: 11 February– 26 March 2022; Study 2 recruitment period: 9 June– 5^th^ July 2022. The studies were preregistered using Aspredicted.com (Study 1: https://aspredicted.org/YQJ_CPG; Study 2: https://aspredicted.org/TQB_T76). Data are freely available at: https://researchbox.org/2404&PEER_REVIEW_passcode=BHMTZT. Both study procedures were approved by the University of Rochester’s Research Subjects Review Board and participants provided electronically written informed consent prior to participation.

#### Sample size determination

The initial preregistered plan was to use multiple regression models. Thus, an *a priori* power analysis using GPower indicated that 528 participants would be required to achieve .80 power using two predictors (resource appraisals and demand appraisals). Additionally, regarding confirmatory factor analysis (CFA), the estimated sample size needed for a 10-item scale with two hypothesized factors is 300 participants [59]. Thus, we set 528 as our minimum sample size.

However, after data collection for both studies was completed, the research team determined that Structural Equation Modeling (SEM) was considered a more suitable fit as it allows for the estimation of measurement error within the models. Using a sample size analysis for SEM, 411 participants would be required to achieve .80 power using two latent variables (resource and demand) and ten observable variables (5 items for each latent variable) for a small-to-medium effect size [60]. Thus, our recruited sample was sufficiently powered to test hypotheses in traditional multiple regression and CFA models, as well as SEM models.

#### Participants

The study included a total of 575 participants recruited from ResearchMatch, Prolific, and a SONA system at a northeast university in the U.S. Participants from Prolific were compensated $9.50/hour and undergraduate students from SONA were awarded extra credit; ResearchMatch participants volunteered without monetary compensation. Demographics of the sample were: *22*.*3%* male, *75*.*3%* female, and *2*.*4%* other; *65*.*4%* White/European American, *5*.*7%* Black/African American, *6*.*9%* LatinX/Hispanic, *18*.*3%* Asian/Asian American, and *3*.*7%* Other: Mean age = 35.52 years (*SD* = 17.93).

#### Data analysis plan

A CFA was conducted to evaluate the theorized factor structure of the observed items (i.e. resource and demand) and to examine the overall fit of the measurement model. SEM was used to test the associations between the resource and demand latent factors and the focal outcome variables; we regressed each outcome variable onto the latent factors in a series of SEM models. Results from multiple regression analyses are included in S2 File. Results from moderation by group identification are included in S3 File. Results of the race-by-gender moderation analyses are presented in S4 File.

#### Data exclusions

Two attention checks were embedded in the questionnaire (e.g., “*It is important that participants read through each question*. *Please select the object that is soft*.” and “*Which sport do you like the most*? *However*, *it is important to read the instructions carefully*, *if you are still reading*, *please ignore the question*, *select ’other*.’”). Participants who responded incorrectly to one or both attention checks were excluded from analyses. We also excluded participants who completed the survey outside of feasible timeframes (< 2 minutes or > 1 hour) (~ 7%).

### Measures

#### Appraisals of social identity

Our new 10-item Identity as a Resource Scale was used to assess appraisals of ethnic-racial identity. The scale includes two subscales: Resource and Demand; sample resource item: “*My ethnic-racial identity is an advantage*;” sample demand item: “*My ethnic-racial identity is a stressor in my life*.” Participants responded using a 1 (Strongly Disagree) to 5 (Strongly Agree) scale. See Table 4 for the final list of items (Cronbach’s α = .88; α = .89, respectively). Details regarding the development of items can be found in S1 File.

#### Self-esteem

Robins and colleagues’ [61] single-item Self-Esteem measure was used to assess participants’ individual self-esteem. It has demonstrated strong convergent validity with other measures of self-esteem (e.g., [62]. The item was assessed on a 1 (Not very true of me) to 7 (Very true of me) scale and was stated as follows: “*I have high self-esteem*.”

The 16-item Collective Self-Esteem Scale [63] was used to assess participants’ collective self-esteem, that is, how they evaluate their social groups; sample item: “*In general*, *I’m glad to be a member of the social groups I belong to*.” Participants responded using a 1 (Strongly Disagree) to 5 (Strongly Agree) scale. After reverse scoring, items were averaged to form the collective self-esteem index (Cronbach’s α = .88).

#### Intergroup outcomes

The 9-item Everyday Discrimination Scale [64] was used to assess participants’ *perceived discrimination*, that is, their day-to-day perceptions of unfair treatment and rejection based on their ethnic-racial identity; sample item: “*You are treated with less respect than other people are*.” Participants responded using a 1 (Never) to 6 (Almost every day) scale (Cronbach’s α = .97).

Using Amodio’s State Affect measure [65], 4 items were adapted to assess participants’ *perceived intergroup anxiety*, that is, their anxiety when interacting with members of other ethnic-racial groups; sample item: “*I feel nervous about interacting with members of other ethnic/racial groups*.” Participants responded using a 1 (Strongly disagree) to 5 (Strongly agree) scale (Cronbach’s α = .97).

Using the General Trust Scale [66], 4 items were adapted and reversed scored to assess participants’ *perceived interracial mistrust*, that is, their level of mistrust regarding members of other ethnic-racial groups; sample item: “*People of other ethnic/racial groups are trustworthy*.” Participants responded using a 1 (Not at all) to 5 (Completely) scale (Cronbach’s α = .95).

The 11-item Behavioral Avoidance Scale [67] was adapted to assess participants’ *behavioral* avoidance, that is, their level of avoidance when interacting with members of other ethnic-racial groups; sample item: “*I avoid spending leisure time with members of other ethnic/racial groups*.” Participants responded using a 1 (Strongly disagree) to 5 (Strongly agree) scale (Cronbach’s α = .97).

#### Distress and coping

The 10-item Perceived Stress Scale [68] was used to assess participants’ reports of *distress* over the past week; sample item: “*In the last week*, *how often have you felt that you were unable to control the important things in your life*?” Participants responded using a 1 (Never) to 5 (Very often) scale. After reverse scoring the 4 negatively worded items, the items were averaged to form the distress index (Cronbach’s α = 0.83).

The 8-item short Grit Scale [69] was used to assess participants’ grit, that is, their goal perseverance and resilience; sample item: “*Setbacks don’t discourage me*.” Participants responded using a 1 (Not like me at all) to 5 (Very much like me) scale. After reverse scoring the 4 negatively worded items, the items were averaged to form the grit index (Cronbach’s α = .77).

### Results

#### Descriptive statistics and correlations

See Table 1 for means, standard deviations, and bivariate correlations of all measures. Resource and demand variables here are averaged composite scores of each subscale.

**Table 1:** Study 1: Means, standard deviations, and bivariate correlations.

	M	SD	1	2	3	4	5	6	7	8	9
1. Resource	3.35	0.88									
2. Demand	1.96	0.88	**-.31** *								
3. Individual Self-Esteem	3.07	1.36	.03	-.06							
4. Collective Self-Esteem	3.64	0.52	**.16** *	**-.19** *	**.26** *						
5. Perceived Discrimination	2.41	0.86	**-.17** *	**.32** *	**-.29** *	**-.29** *					
6. Perceived Intergroup Anxiety	1.89	0.86	**-.12** *	**.33** *	-.05	**-.14** *	**.21** *				
7. Perceived Interracial Mistrust	2.14	.69	**-.25** *	**.21** *	-.08	**-.17** *	**.13** *	**.35** *			
8. Perceived Behavioral Avoidance	1.45	0.56	**-.18** *	**-.18** *	.03	**-.14** *	.03	**.55** *	**-.44** *		
9. Distress	2.94	0.78	-0.04	**.17** *	**-.52** *	**-.32** *	**.44** *	**.13** *	**-.13** *	.01	
10. Grit	3.27	0.72	-0.05	**-.12** *	**.36** *	**.30** *	**-.29** *	**-.17** *	**.12** *	-.05	**-.51** *

*p < .001.

#### CFA

**Fig 1:**
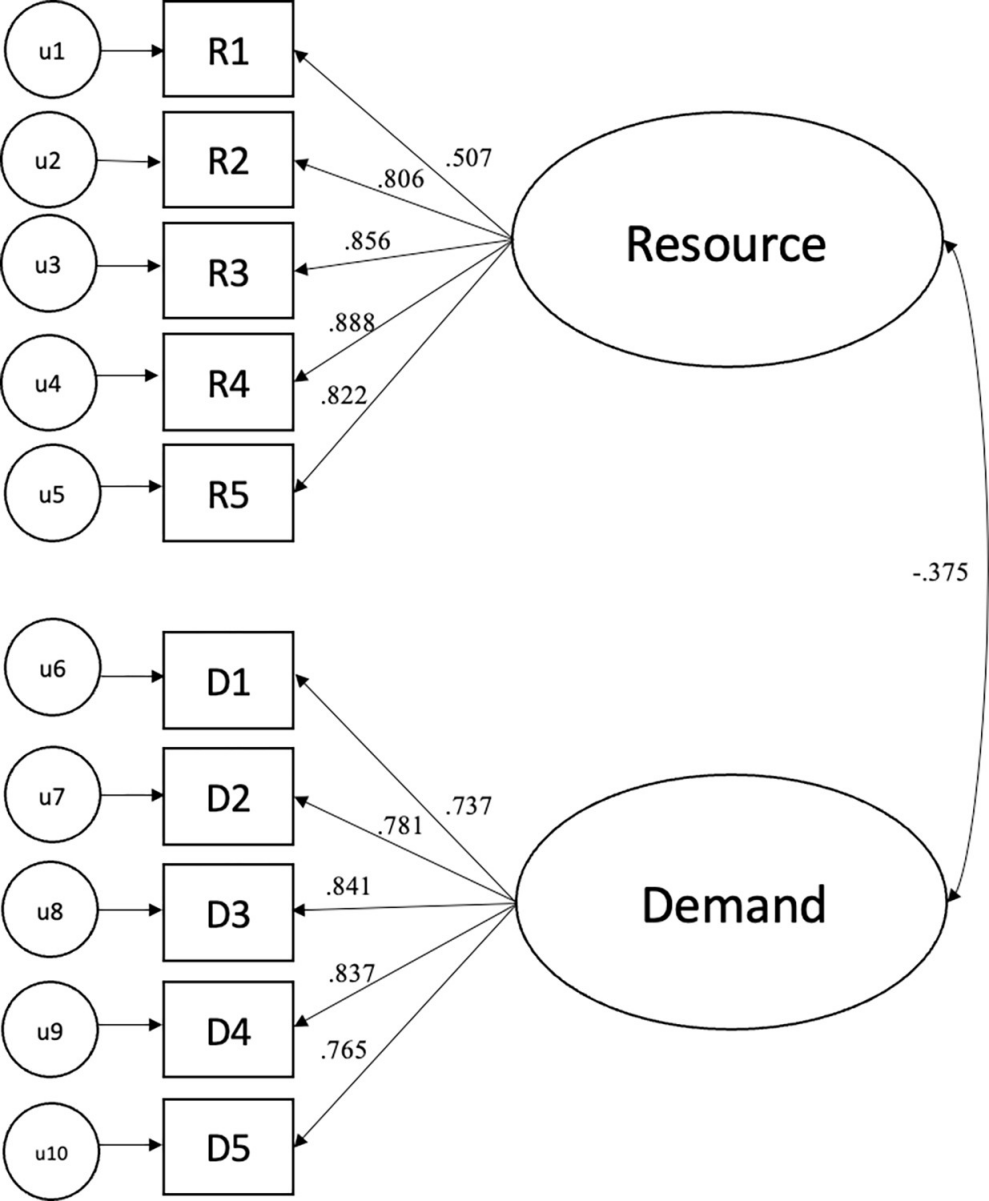
Study 1: Factor loadings for each item and correlation among latent variables.

A CFA was specified in Mplus (Muthen & Muthen, 2008–2019) using the 10 items assessing identity as a resource and a demand. The CFA was specified such that the identity resource items were indicators of the resource latent factor and identity demand items were indicators of the demand latent factor. The model demonstrated adequate fit, X^2^(29, N = 575) = 274.79, *p* < .01, *CFI* = 0.93, *SRMR* = .082 with factor loadings ranging from *λ* = .*65* to .*89* (*p*s < .001, see Fig 1). Resource and demand latent factors were negatively and moderately correlated, r = -.38. The subscales exhibited high levels of internal consistency: Resource Cronbach’s α = .88 and demand Cronbach’s α = .89.

#### SEM

A series of structural equation models (SEMs) were used to examine associations between the resource and demand latent variables and the outcome measures (see Table 2). Full information maximum likelihood estimation was used to handle missing data. Resource and demand latent variables were included as simultaneous independent variables in each model.

**Table 2:** Study 1: Associations between the resource and demand latent variables and outcome measures.

	Resource	Demand
	β	β
Individual Self-Esteem	0.01	-0.05
Collective Self-Esteem	**0.11** *	**-0.14** **
Perceived Discrimination	-0.09	**0.27** ***
Perceived Intergroup Anxiety	-0.03	**0.33** ***
Perceived Interracial Mistrust	**-0.20** ***	**0.16** ***
Perceived Behavioral Avoidance	**-0.13** **	**0.16** ***
Distress	0.01	**0.16** **
Grit	**-0.10** *	**-0.15** **

*p < .05

**p < .01

***p < .001.

*Self-esteem*. Neither resource nor demand appraisals exhibit a significant association with individual self-esteem (*β* = .01, *p* = .917, and *β* = -.05, *p* = .322, respectively). However, resource appraisals were related to greater collective self-esteem, *β* = .11, *p* = .026, whereas higher demand appraisals were related to less collective self-esteem, *β* = -.14, *p* = .01.

*Intergroup outcomes*. Resource appraisals were unrelated to perceived discrimination (*β* = -.09, *p* = .063), whereas demand appraisals were positively related (*β* = .27, *p* < .001). Resource appraisals were also unrelated to intergroup anxiety (*β* = -.03, *p* = .486), whereas demand appraisals were positively related (*β* = .33, *p* < .001). Resource appraisals were negatively related to interracial mistrust (*β* = -.20, *p* < .001), whereas demand appraisals were positively related (*β* = .16, *p* = .001). Resource appraisals were negatively related to behavioral avoidance (*β* = -.13, *p* = .004), whereas demand appraisals were positively related (*β* = .16, *p* = .001).

*Distress and coping*. Resource appraisals were unrelated to distress (*β* = .01, *p* = .861), whereas demand appraisals were positively related (*β* = .16, *p* = .002). Both resource *and* demand appraisals were negatively related to grit (*β* = -.10, *p* = .032; *β* = -.15, *p* = .002, respectively).

### Discussion

The CFA results of Study 1 provide support for the psychometric structure of the novel measure of social identity appraisals (ethnic-racial identity specifically herein) as resources and demands. Furthermore, consistent with hypotheses, individuals who appraised their ethnic-racial identity as a resource reported better self-esteem and improved intergroup outcomes: Higher collective self-esteem and perceived interracial trust, and lower perceived behavioral avoidance. Surprisingly, resource appraisals are also negatively related to grit. Consistent with hypotheses, individuals who perceived their ethnic-racial identity as adding demands to their lives reported worse collective self-esteem, intergroup outcomes (interracial trust, perceived discrimination, intergroup anxiety, and behavioral avoidance), and more distress/worse coping. Interestingly, although individuals may perceive their ethnic-racial identity as a resource, providing advantages or opportunities, this does not necessarily buffer against how they feel about themselves, such as their individual self-esteem. Other factors, such as personal experiences, social feedback, and individual coping mechanisms, are likely play a more significant role in self-esteem [70–72]. Resource and demand components were moderately and negatively correlated, suggesting that they represent related, yet distinct constructs as would be expected based on conceptualizations rooted in BPS models.

Based on fit indices and reliability, and in the interest of economy of respondent effort, we tested a condensed 8-item version of the identity resource and demand scale that omitted the following items: “*My identity is a resource*” and “*My identity is a burden I carry*”.

The pattern of results for the 8-item scale was consistent (i.e., good overall model fit to the data, strong and statistically significant factor loadings, distinct yet moderately negatively correlated latent factors) with that from the 10-item scale, so we proceeded to drop the aforementioned items moving forward in Study 2 in the interest of parsimony.

## Study 2

The focal aim of Study 2 was to replicate and extend the Study 1 findings in an exploratory-confirmatory sequence. Notably, we sought to investigate demographic differences in how social identities are appraised as resources and demands, and to examine whether the 8-item measure assessed the latent constructs of resource and demand across racial groups. As such, we collected data from samples of White and Black participants.

### Method

#### Sample size determination

An *a priori* power analysis using GPower indicated that 725 participants would be required to achieve .80 power using seven predictors (resource, demand, race, resource by demand, race by resource, race by demand, and race by resource by demand) in a multiple regression model. However, as previously stated, SEM was deemed to be a better fit for analyses; therefore, using a sample size calculator for SEM, approximately 652 participants would be required to achieve .80 power using two latent variables (resource and demand) and eight observable variables (4 items for each latent variable) for a small effect size (Soper, 2023).

#### Participants

Study 2 included a sample of White/European Americans (n = 385) and a sample of Black/African Americans (n = 358). Therefore, the overall sample contained 743 participants (*35*.*9%* male, *62*.*2%* female, and .*02%* other) from ResearchMatch and Prolific. ResearchMatch participants volunteered without monetary compensation; Prolific participants were compensated $8.05 per hour. The mean age of the sample was 43.88 years old (*SD* = 16.56). The measures used in this study were the same as those used in Study 1.

#### Measures

See Study 1, with the exception that appraisals of social identity were assessed with the 8-item measure.

#### Data analysis plan and data exclusions

The same data analysis plan and data exclusion criteria used in Study 1 were used in this study. Approximately 8% of participants were excluded from analyses.

### Results

#### Descriptive statistics and correlations

See Table 3 for means, standard deviations, and bivariate correlations. Because this study aimed to examine race similarities and differences, descriptive statistics, and correlations are presented separately for Black and White participants (Black participants are presented above the diagonal, White participants below).

**Table 3:** Study 2: Means, standard deviations, and bivariate correlations split by race.

	M	SD	1	2	3	4	5	6	7	8	9	10	M	SD
1. Resource	3.62	0.95		**-.45** *	**.13** *	**.27** *	**-.12** *	0.02	**.20** *	**.11** *	.01	-.03	2.60	0.84
2. Demand	1.73	0.73	**-.36** *		-.08	**-.27** *	**.42** *	**.29** *	**-.11** *	**.23** *	**-.13** *	**.21** *	2.82	0.94
3. Individual Self-Esteem	3.04	1.36	-.02	-.01		**.39** *	**-.16** *	**-.19** *	.07	-.01	**.55** *	**-.51** *	3.36	1.44
4. Collective Self-Esteem	3.54	0.52	**.16** *	**-.19** *	**.35** *		**.12** *	**.20** *	.06	**.20** *	**.38** *	**-.33** *	3.56	0.59
5. Perceived Discrimination	2.22	0.81	-.05	**.23** *	**-.33** *	**-.24** *		**.42** *	**-.15** *	**.36** *	**-.28** *	**.43** *	2.51	1.05
6. Perceived Intergroup Anxiety	1.74	0.74	-.01	**.19** *	-.06	-.04	.07		**-.20** *	**.56** *	**-.34** *	**.36** *	2.19	1.04
7. Perceived Interracial Mistrust	3.94	0.71	**-.33** *	**.27** *	-.03	**-.13** *	**.15** *	**.37** *		**.18** *	-.10	.02	3.15	0.73
8. Perceived Behavioral Avoidance	1.48	0.59	**-.16** *	**.29** *	.07	.00	-.01	**.61** *	**-.47** *		-.10	**.17** *	1.80	0.76
9. Distress	2.55	0.71	.05	.03	**-.51** *	-.30	**.42** *	.04	-.02	-.05		**-.59** *	2.58	0.78
10. Grit	3.18	0.76	**-.10** *	.09	**.45** *	**.33** *	**-.24** *	**-.10** *	.03	.03	**-.49** *		3.35	0.81

*Note*: Black participants are depicted above the diagonal, and White participants are depicted below the diagonal.

*p < .001.

#### CFA

**Fig 2:**
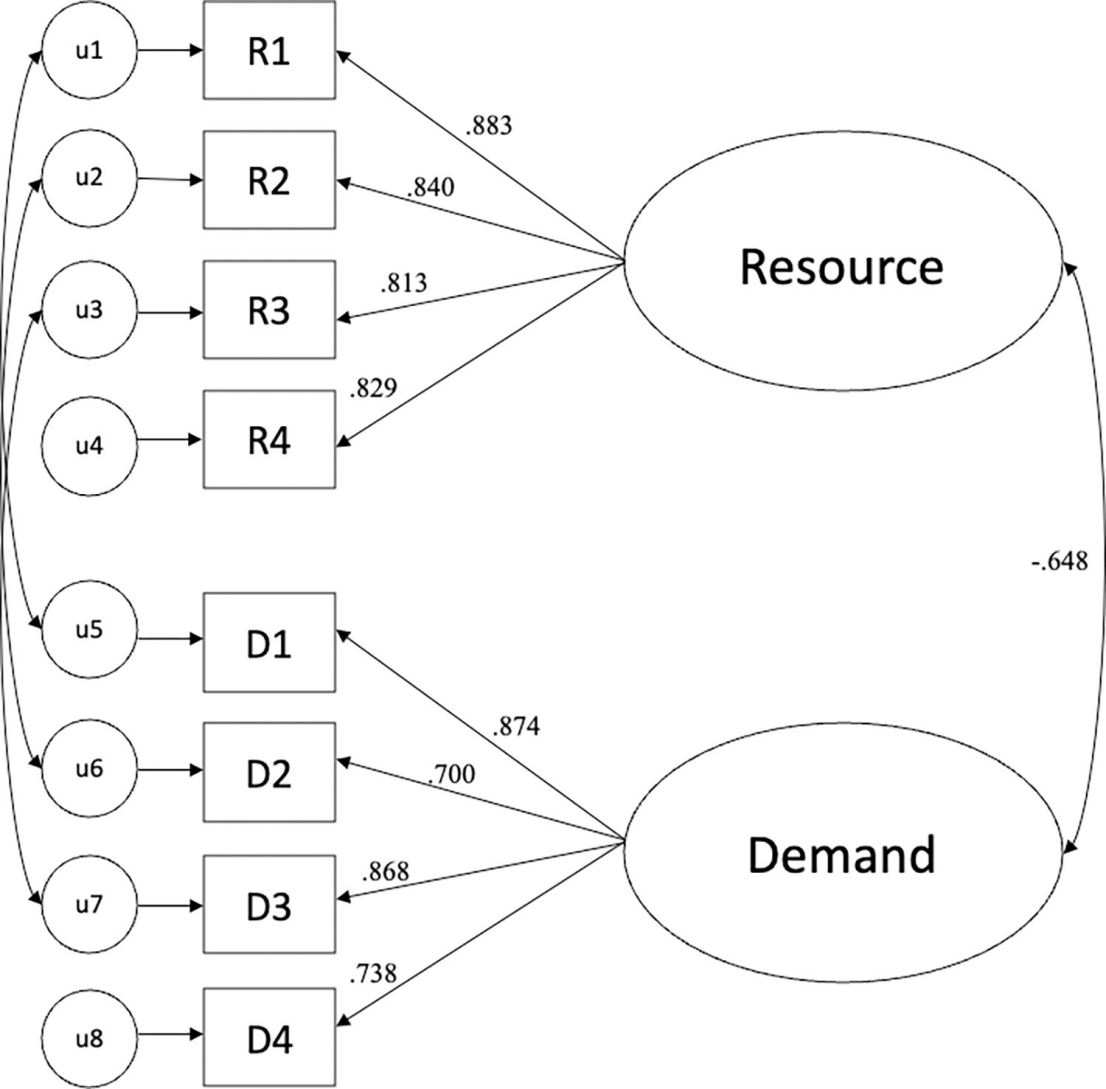
Study 2: Factor loadings for each item and correlation among latent variables.

A CFA with the 8-item scale was conducted using Mplus (Muthen & Muthen, 2008–2019. The model demonstrated good fit, χ^2^(16, N = 743) = 99.60, *p* < .01, *CFI* = 0.98, *SRMR* = .042, with factor loadings ranging from *λ* = .78 to .88 (*p*s < .001; see Fig 2). Again, the two factors were negatively correlated, *r* = -.65, and the subscales showed high levels of internal consistency: Resource Cronbach’s *α* = .91 and Demand Cronbach’s *α* = .89.

#### Mean differences and measurement invariance

Measurement invariance testing was conducted to examine whether the factor loadings for the two latent variables varied significantly across racial groups. The model with freely estimated factor loadings, χ^2^(32) = 99.49, *p* < .001, and the model with constrained factor loadings, χ^2^(38) = 109.41, *p* < .001, were not significantly different, Δχ(6) = 9.92, *p* = .128. Therefore, demonstrating that the factor loadings of the items to their respective latent constructs could be considered equivalent across the two racial groups. This suggests that the constructs may be conceptualized and interpreted in a similar manner between racial groups.

Examining mean differences between racial groups, Black and White participants differed in their appraisals of their ethnic-racial identity (See Table 4). Resource and demand appraisals were averaged into composite scores. White participants (*M* = 3.62, *SD* = .95) viewed their identity as more of a resource relative to Black participants (*M* = 2.60, *SD* = .84), *t*(741) = 15.39, *p* < .001, and Black participants (*M* = 2.82, *SD* = .93) viewed their identity as more of a demand than White participants (*M* = 1.73, *SD* = .73), *t*(741) = -17.68, *p* < .001.

**Table 4:** Study 2: Item means, standard deviations, and independent sample T-tests by race.

	White (n = 385)			Black (n = 358)			
	M	SD	M		SD	t(741)	Cohen’s d
Resource	3.62	0.95	2.60		0.84	**15.39** ***	0.90
My ethnic-racial identity is an advantage.	3.72	1.05	2.59		1.05	**14.72** ***	1.05
My ethnic-racial identity brings me opportunities.	3.58	1.07	2.75		1.03	**10.78** ***	1.05
My ethnic-racial identity helps me.	3.62	1.05	2.89		1.09	**9.23** ***	1.07
My ethnic-racial identity lightens my load.	3.54	1.05	2.17		0.95	**18.56** ***	1.01
Demand	1.73	0.73	2.82		0.93	**-17.68** ***	0.83
My ethnic-racial identity is a disadvantage.	1.74	0.87	3.02		1.17	**-17.07** ***	1.03
My ethnic-racial identity is something I have to overcome.	1.73	0.87	2.77		1.24	**-16.9** ***	1.07
My ethnic-racial identity holds me back.	1.67	0.81	2.75		1.14	**-14.88** ***	0.98
My ethnic-racial identity is a stressor in my life.	1.79	0.89	2.73		1.20	**-12.20** ***	1.05

*Note*: T-tests were conducted to examine whether White and Black participants differed in their appraisal of their ethnic-racial identity as a resource or a demand

*** p < .001.

#### SEM

A series of multiple-group SEMs were estimated to examine the associations among resource and demand latent factors and outcomes, and to determine if these associations varied by race. Given that factor loadings were found to be invariant across racial groups, the measurement portion of the model was constrained across groups and the structural paths were tested for moderation by race. Table 5 presents the chi-square and degrees of freedom for the constrained models (i.e. paths from resource to outcome and from demand to outcome were constrained to equality) and unconstrained models (i.e., paths from resource to outcome and from demand to outcome were freely estimated). Chi-square tests for each set of constrained and unconstrained models are also presented. Table 6 presents the results of the analyses examining the relations between resource and demand appraisals and the outcome variables, broken down by racial group.

**Table 5:** Study 2: Chi-Square differences for moderation by race.

	Constrained Model Fit		Unconstrained Model Fit		Difference in Model Fit	
	df	*χ* ^2^	df	*χ* ^2^	df	*Δχ*
Individual Self-Esteem	58	241.17	56	237.06	2	4.11
Collective Self-Esteem	58	249.14	56	245.28	2	3.86
Perceived Discrimination	58	242.32	56	230.26	2	**12.06** **
Perceived Intergroup Anxiety	58	240.28	56	230.53	2	**9.75** **
Perceived Interracial Mistrust	58	239.56	56	231.36	2	**8.20** **
Perceived Behavioral Avoidance	58	259.61	56	234.58	2	**25.03** **
Distress	58	258.26	56	251.29	2	**6.97** **
Grit	58	243.11	56	233.53	2	**9.58** **

*Note*: Testing White/Black differences in associations between resource and demand appraisals and various outcomes

** p < .05.

**Table 6:** Study 2: Path coefficients by racial group.

		Resource			Demand	
		β	p	β		p
Individual Self-Esteem	White	-0.02	N.S.	-0.01		N.S.
	Black	0.13	N.S.	-0.01		N.S.
Collective Self-Esteem	White	0.10	N.S.	-0.15		*
	Black	0.21	**	-0.16		*
Perceived Discrimination	White	0.08	N.S.	**0.27**		***
	Black	**0.20**	**	**0.58**		***
Perceived Intergroup Anxiety	White	0.09	N.S.	**0.25**		***
	Black	**0.29**	***	**0.47**		***
Perceived Interracial Mistrust	White	**-0.25**	***	**0.20**		***
	Black	**-0.23**	**	-0.02		N.S.
Perceived Behavioral Avoidance	White	-0.03	N.S.	**0.32**		***
	Black	**0.38**	***	**0.44**		***
Distress	White	0.08	N.S.	0.05		N.S.
	Black	**0.15**	*	**0.32**		***
Grit	White	-0.08	N.S.	0.08		N.S.
	Black	-0.10	N.S.	**-0.20**		**

*Note*: Results from unconstrained models; Individual Self-Esteem and Collective Self-Esteem are not significantly moderated by race

*p < .05

**p < .01

***p < .001.

*Self-esteem*. Associations between individual self-esteem and resource and demand appraisals did not significantly differ for White and Black participants (Δχ(2) = 4.11, *p* = .128). Likewise, no significant racial differences were observed for associations between appraisals and collective self-esteem (Δχ(2) = 3.86, *p* = .145). These were the only outcomes that did not evidence significant moderation by race.

When collapsed across race, similar to Study 1, resource and demand appraisals were not significantly correlated with individual self-esteem, (*β* = .01, *p* = .933, and *β* = .03, *p* = .569, respectively). Resource appraisals were related to greater collective self-esteem, *β* = .11, *p* = .039, whereas higher demand appraisals were related to less collective self-esteem, *β* = -.12, *p* = .036.

*Intergroup outcomes*. White and Black participants exhibited different associations between resource and demand appraisals and perceived discrimination (Δχ (2) = 12.06): While resource appraisals were unrelated to perceived discrimination for White participants (*β* = .08, *p* = .194), for Black participants the association was (surprisingly) positive (*β* = .20, *p* < .005). Demand appraisals were positively related to perceived discrimination for both White and Black participants (*β* = .27, *p* < .001, and *β* = .58, *p* < .001, respectively).

White and Black participants exhibited different associations between resource and demand appraisals and perceived intergroup anxiety (Δχ (2) = 9.75): While resource appraisals were unrelated to perceived intergroup anxiety for White participants (*β* = .09, *p* = .115), for Black participants the association was (again, surprisingly) positively related (*β* = .29, *p* < .001).

Demand appraisals were positively related to perceived intergroup anxiety for both White and Black participants (*β* = .25, *p* < .001, and *β* = .47, *p* < .001, respectively).

White and Black participants exhibited different associations between resource and demand appraisals and perceived interracial mistrust (Δχ (2) = 8.20): Resource appraisals were negatively related to perceived interracial mistrust for both White and Black participants (*β* = -.25, *p* < .001, and *β* = -.23, *p* = .002, respectively). However, demand appraisals for White participants were negatively related to perceived interracial trust (*β* = -.20, *p* < .001), but for Black participants the two were unrelated (*β* = .02, *p* = .845).

White and Black participants exhibited different associations between resource and demand appraisals and perceived behavioral avoidance (Δχ (2) = 25.03): While resource appraisals were unrelated to perceived behavioral avoidance for White participants (*β* = -.03, *p* = .608), for Black participants the association was positively related (*β* = .38, *p* < .001).

Demand appraisals were positively related to perceived behavioral avoidance for both White and Black participants (*β* = .32, *p* < .001, and *β* = .44, *p* < .001, respectively).

*Distress and coping*. White and Black participants exhibited different associations between resource and demand appraisals and distress (Δχ (2) = 6.97): While resource appraisals were unrelated to distress for White participants (*β* = .08, *p* < .167), for Black participants the association was positively related (*β* = .15, *p* = .043). Although demand appraisals were unrelated to distress for White participants (*β* = .05, *p* = .461), for Black participants the association was positively related (*β* = .32, *p* < .001).

White and Black participants exhibited different associations between resource and demand appraisals and grit (Δχ (2) = 9.58): Resource appraisals were unrelated to grit for both White and Black participants (*β* = -.08, *p* < .189, and *β* = -.10, *p* = .172, respectively). Although demand appraisals were unrelated to grit for White participants (*β* = .08, *p* = .183), for Black participants the association was negatively related (*β* = -.20, *p* = .01).

### Discussion

Results from Study 2 were broadly consistent with hypotheses and the data observed in Study 1. Individuals who perceived their ethnic-racial identity as a resource reported better social and intergroup outcomes while those who appraised their identity as a demand reported worse social and intergroup outcomes. Study 2 sought to further investigate these associations by testing differences as a function of race, specifically testing whether White and Black participants appraised their ethnic-racial identities differently. Black participants on average perceived their ethnic-racial identity as more demanding than their White counterparts and reported worse social and intergroup outcomes relative to Whites. Among Black participants, and contrary to predictions, appraisals of their identity as a resource was associated with higher discrimination, intergroup anxiety, and behavioral avoidance; whereas among White participants, appraisals of their ethnic-racial identity as a resource were unrelated to those outcomes. White and Black participants who perceived their ethnic-racial identity as more demanding reported higher discrimination, intergroup anxiety, behavioral avoidance, and lower interracial trust.

These findings suggest that appraisals of social identities can impact perceptions of various social and intergroup outcomes. However, certain social identities, like ethnic-racial identity, can pose disadvantages for individuals irrespective of their personal perceptions, and the detrimental effects can intensify when these identities are perceived as more demanding. The way others or the world perceives one’s identity can significantly shape one’s social and intergroup experiences. Consider an individual who identifies positively with their ethnic-racial identity. Despite their affirming personal perceptions of this identity, societal stereotypes and biases may lead them to view their identity as more demanding. In such cases, external perspectives can significantly shape their experiences, potentially leading to adverse outcomes.

## General discussion

### Key findings and interpretation

This study is the first examination of how individuals appraise their social identities as resources that support goal attainment and demands that present hurdles to be overcome. Study 1 established the reliability and validity of a novel measure and tested how resource and demand appraisals mapped onto various social and intergroup outcomes. Individuals appraising their ethnic-racial identity as a helpful resource reported higher collective self-esteem, and lower discrimination, interracial mistrust, intergroup anxiety, and behavioral avoidance. On the other hand, individuals who perceived their ethnic-racial identity as introducing demands in their lives reported higher discrimination, intergroup anxiety, interracial mistrust, and behavioral avoidance, as well as lower collective self-esteem. Notably, while resource and demand appraisals were negatively correlated, they emerged as independent constructs, suggesting that these appraisal categories can operate independently, consistent with conceptualizations of stress appraisals in the BPS model of challenge and threat [2].

### Replication and extension (study 2)

Study 2 extended and replicated findings from Study 1. To do so, well-powered samples of both Black and White Americans were recruited, and ethnic-racial identity was examined as a moderator. Notably, Black Americans who appraised their identity as more demanding reported the worst social and intergroup outcomes, which may not be surprising given that Black Americans have historically faced more prejudice and discrimination compared to White Americans (e.g. [73–76]). Interestingly, Black individuals who appraised their identity as a resource reported more, not less, perceived discrimination, intergroup anxiety, and behavioral avoidance. While future research is needed to explore the mechanisms underlying this association, it is possible that racial identity plays a more central role in the self-identity of Black Americans who view their racial identity as a greater resource compared to those who do not. In other words, when individuals perceive benefits from their racial identity, it is more likely to be more strongly integrated into their self-concept [77].

This finding underscores the distinct nature of individual-level resource appraisals compared to group-level constructs like group identification or collective self-esteem, which focuses on group connection and evaluation. Resource appraisals, as operationalized here, capture individual-level perceptions of one’s identity as personally beneficial. For example, resource appraisals were not correlated with group identification in either study, whereas demand appraisals showed a small positive correlation with group identification in Study 1 (*β* = .11). This highlights the self-referential and personal nature of identity appraisals, suggesting they function independently of broader group-level constructs.

At the same time, the social cure literature suggests that identity advantages also encompass social support and belonging, which were not explicitly measured here [18, 19, 30]. Moreover, the data from Study 2 suggests individuals holding potentially stigmatized identities may be more likely to exhibit more negative intergroup outcomes regardless of how they appraise those identities—that is, the coping resources conferred by one’s identity may not be sufficient to protect against negative intergroup outcomes.

### Psychometric validation

Although Black individuals endorsed higher levels of demand appraisals and lower levels of resource appraisals relative to White individuals in this research, the individual scale items did not differentially relate to the resource and demand constructs for people holding different ethnic-racial identities, indicating that White and Black individuals perceived the items in the scale similarly. This suggests that the scale is a reliable measure for assessing identity appraisals at least across these two prominent racial categories in American society.

### Implications for health and well-being

More broadly, the pattern of data observed here suggests that identity appraisals correlate with notable intergroup psychological outcomes with important implications for health and well-being [64, 76, 78–81]. For instance, the more participants view their ethnic-racial identity as a demand to be overcome, the greater the perceived distress in their lives. This has notable health implications given research demonstrating links among perceived distress and negative health outcomes [82–84]. Identity appraisals may lead to variations in stress levels, coping mechanisms, and compliance with medical advice, ultimately contributing to disparities in health outcomes among diverse populations [48, 85].

Perceived discrimination is also associated with increased substance use among Black individuals, and researchers often investigate the degree to which perceptions of discrimination, both racial and non-racial, serve as stressful life experiences that predict of negative health outcomes [86–89]. In addition, intergroup anxiety yields numerous adverse consequences [90–92]. This affective process can hinder performance, redirect attention toward negative cues, and anticipate adverse effects on biological functioning [39, 93, 94]. Prior to these studies, research had yet to elucidate how individual-level social identities were appraised as resources or demands in the context of stress models such as the BPS model of challenge and threat.

## Limitations and future directions

The present research suggests that social identity appraisals can have important influences on social experiences, intergroup behaviors, and well-being. Yet, the existing social identity and stress literature lacks such a measure. While the current scale lends itself to the examination of myriad social groups, the present work focused on individuals broadly (Study 1) and Black and White individuals specifically (Study 2). Future empirical work would benefit from examining other social identities, including but not limited to religion, sexual orientation, and gender, as well as more nuanced, intersectional identities such as race plus social class.

The U.S. sample also cannot speak to the experiences of Black or other stigmatized individuals in other cultural settings. Identity appraisals may vary significantly based on the predominant racial or ethnic composition of a given country. For example, in nations where Black individuals constitute the majority (e.g., certain African countries), the dynamics of identity appraisals and the psychological outcomes we examined would likely differ from those observed in the U.S. Even among Western cultures (e.g., U.S. vs. European countries) there is substantial heterogeneity in marginalization of groups. In addition, research on immigrant populations has shown that ethnic identity and acculturation strategies are crucial factors influencing adjustment to new environments, with significant implications for psychological well-being, particularly in contexts of minority status and contact with host populations [95]. Thus, there is a need for future research to explore how stress and identity dynamics may operate in diverse, global samples to gain a more comprehensive understanding of how appraisals of identity shape stress and health processes.

Another possible limitation of our study is the recruitment of university students and people from the general population. This mixed sample, while more diverse than a single recruitment approach, has the potential to introduce variability tied to developmental processes, socioeconomic backgrounds, educational experiences, and social contexts. Future research could seek to better capture and test for demographic moderators (even with a U.S. sample) and expand recruitment to children and older adults. For instance, it would be interesting to examine how identity appraisals develop and whether these remain stable across time and whether different socioeconomic groups demonstrate different trajectories.

Moreover, it’s crucial to acknowledge that individuals hold multiple social identities and may perceive advantages stemming from one identity (e.g., being White) while simultaneously perceiving disadvantages based on another identity (e.g., identifying as a sexual minority). For example, individuals with invisible disabilities face unique challenges, as their conditions—and linked identities—are not immediately apparent, which can lead to exclusion and discrimination in both social and professional environments [96]. These initial studies focused on ethnic-racial identity due to its visibility and substantial attention race receives in American culture, serving as a foundation for the scale. The scale’s generative nature allows for a deeper exploration of perceptions across various types of identities. Therefore, the authors intend to validate the scale across diverse identities and populations in future research.

Additionally, because the current research is correlational and cross-sectional, it is limited in allowing interpretations regarding causality and directionality. While using self-report measures is beneficial to understanding the initial associations between the scale and various psychological and intergroup outcomes, it also has its shortcomings. To further this line of research, we are currently examining how the present scale maps onto physiological stress responses associated with challenge and threat. This will assist in allowing a test of the causal effects of identity appraisals on stress responses and objective indicators of health/well-being. Lastly, an additional avenue for future research is to assess how fluctuations in individuals’ perceptions of resource and demand appraisals relate to changes in their mental health and resilience over time.

In the context of race and ethnicity, the appraisal scale developed here offers a unique perspective to understand how individuals perceive their identity (i.e., as resourceful or demanding). Beyond the BPS model of challenge and threat, other research traditions helped lay the groundwork for the development of this measure. For instance, the Multidimensional Model of Racial Identity (MMRI; [77]), multigroup model of ethnic identity, and Nigrescence models suggest what racial/ethnic identity means to individuals from marginalized backgrounds. Moreover, general Collective Self-Esteem (CSE) and Multigroup Ethnic Identity (MEIM) measures examine shared elements across groups, such as group-identification, sense of belonging, and attitudes toward one’s group [63, 97]. More specific African-American identity, the Multidimensional Model of Racial Identity (MMRI) advocates for multiple dimensions of identity (e.g., [77, 98]. Building on these frameworks and integrating concepts with the BPS model of challenge and threat, the measure presented herein demonstrates how cognitive appraisals of ethnic-racial identity as resources and demands shapes stress and psychological processes.

This research provides valuable insights into the dynamics of identity appraisals among Black and White participants in the U.S.. While race is a prominent topic in U.S. culture, in other countries, factors such as religion, ethnicity, or immigration status often hold greater societal significance. For instance, in countries with predominantly White populations that also have substantial stigmatized minority groups—such as East Asian, Pakistani, or Romanian communities—different social dynamics may emerge, potentially leading to distinct identity appraisals and psychological outcomes. Given the variability in the importance of race and other identity factors across different cultural contexts, future research should investigate how these dynamics operate in diverse international settings. Understanding the nuances of identity appraisals in various cultural contexts will enhance the generalizability of our findings and contribute to a more comprehensive understanding of social identity processes.

While this study provides a novel lens through which identity as a resource can be understood, it has limitations. The operationalization of resource appraisals focuses on individual-level benefits, such as goal facilitation, rather than collective or group-level aspects like social support or belonging. Thus, this narrow focus, while helpful for understanding how identity appraisals shape individual level stress responses, cannot fully capture the multifaceted ways in which identity can possibly function as a resource (e.g., [18, 19, 30, 99, 100]). Future research may seek to better understand how these collective resources combine with personal identity appraisals.

Lastly, the present study centers on perceived or subjective outcomes. Future work would benefit from documenting behavioral changes stemming from identity appraisals. For example, does endorsing an identity-as-resource frame buffer against instances of discrimination for disadvantaged groups? If so, future efforts in developing interventions could prove highly advantageous for health and well-being.

## Conclusion

In conclusion, the present research provides valuable insights into how individuals appraise their social identities as resources and/or demands, and the association among identity appraisals and psychological outcomes. By demonstrating the psychometric validity of a novel measure of identity appraisals and highlighting its predictive utility across self-based and intergroup outcomes, this work advances the understanding of how social identities influence psychological functioning.

The study’s broader implications extend to health, psychological functioning, and well-being, emphasizing the need for nuanced approaches to identity research that consider both resource and demand perspectives. Specifically, the findings underscore the dual nature of social identities: while appraising an identity as a resource may enhance resilience and promote positive intergroup interactions, perceiving it as a demand can exacerbate stress, anxiety, and avoidance behaviors. These effects are particularly salient for individuals with potentially stigmatized identities, such as racial minorities, for whom even resource appraisals may coexist with heightened discrimination and social challenges.

Furthermore, this research highlights the importance of situational and contextual factors in shaping identity appraisals and their downstream effects. This suggests that the appraisal of identity as either a resource or demand may not only shape immediate emotional responses but also influence broader social and coping dynamics, reinforcing the need for identity-reframing interventions to improve well-being [101]. Future research should explore the mechanisms underlying these appraisals, including how external cues, social environments, and cultural narratives influence whether an identity is perceived as a resource or demand. In sum, this research contributes to a better understanding of the psychological dynamics associated with social identities, providing a foundation for future studies to further explore the interplay between identity appraisals and diverse outcomes. By integrating these perspectives, this work offers a pathway toward more comprehensive theories and process-focused interventions in identity and intergroup relations.

## Supporting information

S1 File Development of items. (DOCX)

S2 File Regression analyses. (DOCX)

S3 File Moderation by group identification. (DOCX)

S4 File Moderation by race and gender. (DOCX)
