# Supplementary material for: Identity as a resource or a demand
Source: PLoS One. 2025 Jan 28;20(1):e0318449. doi: 10.1371/journal.pone.0318449 (PMC11774354; doi:10.1371/journal.pone.0318449)
Supplement: S1 File — (DOCX) [file pone.0318449.s001.docx]

**S1 File. Development of Items**

In this section, we provide information about two pilot studies conducted prior to Study 1. This includes a list of the items and the correlations between resource and demand appraisals with the tested outcomes.

The Authors conducted two pilot studies on Amazon Mechanical Turk (MTurk) prior to determining the final items and fit of scale. Pilot I study (N = 203) included 9 items for the resource subscale and 9 items for the demand subscale (Resource Cronbach’s α = .95 and demand Cronbach’s α = .96). Pilot II study (N = 196) included 4 items for the resource subscale and 4 items for the demand subscale (Resource Cronbach’s α = .85 and demand Cronbach’s α = .94). Participants from both samples were majority White/European American. Based on preliminary factor analysis and the aim for a concise and efficient scale, the authors opted for the 8-item scale.

**Pilot I Items**

| **Resource**  My ethnic-racial identity is a resource in my life.  My ethnic-racial identity is an advantage.  My ethnic-racial identity brings me opportunities.  My ethnic-racial identity helps me.  My ethnic-racial identity lightens my load.  My ethnic-racial identity is a privilege.  My ethnic-racial identity is a strength.  My ethnic-racial identity is a source of pride.  My ethnic-racial identity benefits me.  **Demand**  My ethnic-racial identity is a stressor in my life.  My ethnic-racial identity is a disadvantage.  My ethnic-racial identity is something I have to overcome.  My ethnic-racial identity holds me back.  My ethnic- racial identity is a burden I carry.  My ethnic-racial identity is a curse.  My ethnic-racial identity is a weakness.  My ethnic-racial identity is a source of shame.  My ethnic-racial identity gets in the way. |
| --- |

*Pilot I: Associations Between the Resource and Demand Composite Variables and Outcomes Measures*

|  | Resource | Demand |
| --- | --- | --- |
|  | β | β |
| Individual Self-Esteem | **.17*** | -.01 |
| Collective Self-Esteem | **.24***** | **-.48***** |
| Perceived Discrimination | **.28***** | **.69***** |
| Grit | -.12 | **-.34***** |

*

p < .05. **p < .01. ***p < .001.

**Pilot II Items**

| **Resource**  My ethnic-racial identity is a resource in my life.  My ethnic-racial identity is an advantage.  My ethnic-racial identity brings me opportunities.  My ethnic-racial identity helps me.  **Demand**  My ethnic-racial identity is a stressor in my life.  My ethnic-racial identity is a disadvantage.  My ethnic-racial identity is something I have to overcome.  My ethnic-racial identity holds me back. |
| --- |

*Pilot II: Associations Between the Resource and Demand Composite Variables and Outcomes Measures*

|  | Resource | Demand |
| --- | --- | --- |
|  | β | β |
| Individual Self-Esteem | **.24***** | **.15*** |
| Collective Self-Esteem | **.16*** | **-.44***** |
| Perceived Discrimination | **.26***** | **.69***** |
| Perceived Intergroup Anxiety | **.16*** | **.77***** |
| Perceived Interracial Mistrust | **-.37***** | .02 |
| Perceived Behavioral Avoidance | **.25***** | **.77**** |
| Distress | .04 | **.24***** |
| Grit | -.05 | **-.24***** |

*p < .05. **p < .01. ***p < .001.
