# Supplementary material for: Identity as a resource or a demand
Source: PLoS One. 2025 Jan 28;20(1):e0318449. doi: 10.1371/journal.pone.0318449 (PMC11774354; doi:10.1371/journal.pone.0318449)
Supplement: S2 File — (DOCX) [file pone.0318449.s002.docx]

**S2 File. Regression Analyses**

In this section, we present the regression results examining the relationships between resource and demand appraisals and the outcome measures, as regression analysis was the pre-registered analytical approach for the study. Data were analyzed using IBM SPSS Statistics (version 29).

|  | Resource | Demand |
| --- | --- | --- |
|  | β | β |
| Individual Self-Esteem | .02 | -.06 |
| Collective Self-Esteem | **.11**** | **-.16***** |
| Perceived Discrimination | -.08 | **.29***** |
| Perceived Intergroup Anxiety | -.02 | **.33***** |
| Perceived Interracial Mistrust | **-.20***** | **.15***** |
| Perceived Behavioral Avoidance | **-.14**** | **.14**** |
| Distress | .01 | **.17***** |
| Grit | **-.09*** | **-.15***** |

*Study 1: Associations Between the Resource and Demand Composite Variables and Outcomes Measures*

*p < .05. **p < .01. ***p < .001.

|  | Resource | Demand |
| --- | --- | --- |
|  | β | β |
| Individual Self-Esteem | -.00 | .02 |
| Collective Self-Esteem | **.10*** | **-.13**** |
| Perceived Discrimination | **.08*** | **.41***** |
| Perceived Intergroup Anxiety | **.10*** | **.39***** |
| Perceived Interracial Mistrust | **-.32***** | **.22***** |
| Perceived Behavioral Avoidance | .08 | **.38***** |
| Distress | **.10*** | **.18***** |
| Grit | **-.12**** | -.03 |

*Study 2: Associations Between the Resource and Demand Composite Variables and Outcomes Measures Collapsed Across Race*

*p < .05. **p < .10. ***p < .001.
