# Supplementary material for: Identity as a resource or a demand
Source: PLoS One. 2025 Jan 28;20(1):e0318449. doi: 10.1371/journal.pone.0318449 (PMC11774354; doi:10.1371/journal.pone.0318449)
Supplement: S3 File — (DOCX) [file pone.0318449.s003.docx]

**S3 File. Moderation by Group Identification.**

In this section, we present the results of the moderation analysis by group identification on the relationship between resource and demand appraisals and the tested outcomes, collapsed across race for both studies. The analyses were conducted using SEM models in Mplus. In Study 1, group identification moderated the relationship between resource appraisals and collective self-esteem. In Study 2, group identification moderated the relationship between resource appraisals and both intergroup anxiety and behavioral avoidance, as well as the relationship between demand appraisals and perceived discrimination.

*Study 1: Moderation by Group Identification*

|  | Resource | Demand | Group Identification | Resource* Group ID | Demand* Group ID |
| --- | --- | --- | --- | --- | --- |
|  | β | β | β | β | β |
| Individuals Self-Esteem | -.01 | -.08 | **.21***** | -.03 | -.03 |
| Collective Self-Esteem | .06 | **-.20***** | **.47***** | **.09**** | -.02 |
| Perceived Discrimination | -.05 | **.33***** | **-.20***** | -.03 | .00 |
| Perceived Intergroup Anxiety | -.02 | **.34***** | **.15***** | .02 | -.04 |
| Perceived Interracial Mistrust | **-.21***** | **.14**** | .05 | .01 | -.05 |
| Perceived Behavioral Avoidance | **-.15**** | **.13**** | **.10*** | .00 | -.05 |
| Distress | .04 | **.20***** | **-.20***** | .01 | -.01 |
| Grit | -.12 | **-.18***** | **.18***** | .01 | -.04 |

*p < .05. **p < .01. *** p < .001.

Group ID moderation (Resource on Collective Self-Esteem)

When group identification is at mean level, resource appraisals were unrelated to collective self-esteem (β = .07, SE = .04, p = .100). When group identification is high, resource appraisals were positively related to collective self-esteem (β = 0.16, SE = .06, p = .005). When group identification is low, resource appraisals were unrelated to collective self-esteem (β = -0.02, SE = .05, p = .675).

|  | Resource | Demand | Group Identification | Resource* Group ID | Demand* Group ID |
| --- | --- | --- | --- | --- | --- |
|  | β | β | β | β | β |
| Individuals Self-Esteem | -.02 | -.02 | **.31***** | .02 | .02 |
| Collective Self-Esteem | .08 | **-.21***** | **.58***** | .00 | .04 |
| Perceived Discrimination | **.17**** | **.51***** | **-.12***** | -.04 | **.07*** |
| Perceived Intergroup Anxiety | **.17**** | **.46***** | .01 | **.07*** | -.001 |
| Perceived Interracial Mistrust | **-.32***** | **.21***** | **.09**** | .05 | -.04 |
| Perceived Behavioral Avoidance | **.14**** | **.43***** | **.07*** | **.06*** | -.02 |
| Distress | **.16**** | **.25***** | **-.30***** | .00 | .008 |
| Grit | **-.14**** | -.09 | **.33***** | .03 | -.04 |

*Study 2: Moderation by Group Identification Collapsed Across Race*

*p < .05. **p < .01, *** p < .001

Group ID moderation (Resource on Perceived Intergroup Anxiety)

When group identification is at mean level, resource appraisals were positively related to intergroup anxiety (β = 0.18, SE = .05, p < .001). When group identification is high, resource appraisals were positively related to intergroup anxiety (β = 0.25, SE = .06, p < .001). When group identification is low, resource appraisals were positively related to intergroup anxiety (β = .12, SE = .06, p = .042).

Group ID moderation (Resource on Perceived Behavioral Avoidance)

When group identification is at mean level, resource appraisals were positively related to behavioral avoidance (β = 0.15, SE = .05, p = .004). When group identification is high, resource appraisals were positively related to behavioral avoidance (β = 0.21, SE = .06, p = .001). When group identification is low, resource appraisals were unrelated to behavioral avoidance (β = 0.08, SE = .06, p = .145).

Group ID moderation (Demand on Perceived Discrimination)

When group identification is at mean level, demand appraisals were positively related to discrimination (β = 0.51, SE = .05, p < .001). When group identification is high, demand appraisals were positively related to discrimination (β = 0.58, SE = .06, p < .001). When group identification is low, demand appraisals were positively related to discrimination (β = 0.44, SE = .06, p < .001).
