# Supplementary material for: Identity as a resource or a demand
Source: PLoS One. 2025 Jan 28;20(1):e0318449. doi: 10.1371/journal.pone.0318449 (PMC11774354; doi:10.1371/journal.pone.0318449)
Supplement: S4 File — (DOCX) [file pone.0318449.s004.docx]

**S4 File. Moderation by Race and Gender.**

In this section, we present the results of the moderation analysis by race and gender on the relationship between resource and demand appraisals and the tested outcomes. The analyses were conducted using the R package *lavaan*. In Study 1, collective self-esteem was the only outcome moderated by both race and gender. In Study 2, perceived discrimination, behavioral avoidance, and distress were moderated by race and gender.

*Study 1: Chi-Square Differences for Moderation by Race and Gender.*

|  | Constrained Model Fit | | Unconstrained Model Fit | | Difference in Model Fit | |
| --- | --- | --- | --- | --- | --- | --- |
|  | df | 𝒳² | df | 𝒳² | df | 𝞓𝒳 |
| Individual Self-Esteem | 154 | 440.43 | 148 | 434.92 | 6 | 5.52 |
| Collective Self-Esteem | 154 | 457.54 | 148 | 428.62 | 6 | **28.91**** |
| Perceived Discrimination | 154 | 434.19 | 148 | 425.03 | 6 | 9.16 |
| Perceived Intergroup Anxiety | 154 | 417.63 | 148 | 407.58 | 6 | 10.06 |
| Perceived Interracial Mistrust | 154 | 418.11 | 148 | 412.07 | 6 | 6.05 |
| Perceived Behavioral Avoidance | 154 | 424.82 | 148 | 419.83 | 6 | 4.99 |
| Distress | 154 | 427.94 | 148 | 424.56 | 6 | 3.38 |
| Grit | 154 | 407.72 | 148 | 397.73 | 6 | 9.99 |

*p < .05. **p < .01. ***p < .001.

*Study 1: Chi-Square Differences for Moderation by Gender.*

|  |  | Constrained Model Fit | | Unconstrained Model Fit | | Difference in Model Fit | |
| --- | --- | --- | --- | --- | --- | --- | --- |
|  |  | df | 𝒳² | df | 𝒳² | df | 𝞓𝒳 |
| Collective Self-Esteem | Females | 76 | 282.91 | 74 | 265.12 | 2 | **17.79**** |
|  | Males | 76 | 168.67 | 74 | 163.50 | 2 | 5.17 |

*p < .05. **p < .01. ***p < .001.

*Study 1: Path coefficients by Racial and Gender Group.*

|  |  |  | Resource | | | Demand | |
| --- | --- | --- | --- | --- | --- | --- | --- |
|  |  |  | β | p | β | | p |
| Collective Self-Esteem | Females | White | 0.04 | N.S. | -0.12 | | 0.05 |
|  |  | Minoritized | **0.41** | ******* | -0.08 | | N.S. |
|  | Males | White | -0.05 | N.S. | -0.19 | | N.S. |
|  |  | Minoritized | 0.29 | N.S. | **-0.29** | | ******* |

Note Sample includes 128 males (60.9% White and 39.1% Minoritized) and 433 females (67.2% White and 32.9% Minoritized). Results from unconstrained models; *p < .05. **p < .01. ***p < .001.

*Study 2: Chi-Square Differences for Moderation by Race and Gender.*

|  | Constrained Model Fit | | Unconstrained Model Fit | | Difference in Model Fit | |
| --- | --- | --- | --- | --- | --- | --- |
|  | df | 𝒳² | df | 𝒳² | df | 𝞓𝒳 |
| Individual Self-Esteem | 94 | 162.49 | 88 | 157.10 | 6 | 5.39 |
| Collective Self-Esteem | 94 | 170.51 | 88 | 166.15 | 6 | 4.37 |
| Perceived Discrimination | 94 | 171.92 | 88 | 156.90 | 6 | **15.02**** |
| Perceived Intergroup Anxiety | 94 | 153.90 | 88 | 142.87 | 6 | 11.04 |
| Perceived Interracial Mistrust | 94 | 157.05 | 88 | 145.75 | 6 | 11.30 |
| Perceived Behavioral Avoidance | 94 | 168.10 | 88 | 143.33 | 6 | **24.78**** |
| Distress | 94 | 187.64 | 88 | 173.58 | 6 | **14.06**** |
| Grit | 94 | 162.59 | 88 | 152.99 | 6 | 9.60 |

*p < .05. **p < .01. ***p < .001.

*Study 2: Chi-Square Differences for Moderation by Gender.*

|  |  | Constrained Model Fit | | Unconstrained Model Fit | | Difference in Model Fit | |
| --- | --- | --- | --- | --- | --- | --- | --- |
|  |  | df | 𝒳² | df | 𝒳² | df | 𝞓𝒳 |
| Perceived Discrimination | Females | 46 | 102.03 | 44 | 93.91 | 2 | **8.12**** |
|  | Males | 46 | 69.55 | 44 | 62.98 | 2 | **6.57**** |
| Perceived Behavioral Avoidance | Females | 46 | 87.39 | 44 | 80.95 | 2 | **6.44**** |
|  | Males | 46 | 72.64 | 44 | 62.38 | 2 | **10.26**** |
| Distress | Females | 46 | 110.26 | 44 | 100.33 | 2 | **9.93**** |
|  | Males | 46 | 74.14 | 44 | 73.25 | 2 | 0.90 |

*p < .05. **p < .01. ***p < .001.

*Study 2: Path coefficients by Racial and Gender Group.*

|  |  |  | Resource | | Demand | |
| --- | --- | --- | --- | --- | --- | --- |
|  |  |  | β | p | β | p |
| Perceived Discrimination | Females | White | 0.10 | N.S. | **0.25** | ****** |
|  |  | Black | 0.21 | N.S. | **0.65** | ******* |
|  | Males | White | -0.06 | N.S. | **0.30** | ****** |
|  |  | Black | **0.31** | ***** | **0.65** | ******* |
| Perceived Behavioral Avoidance | Females | White | -0.03 | N.S. | **0.21** | ******* |
|  |  | Black | **0.22** | ****** | **0.30** | ****** |
|  | Males | White | 0.04 | N.S. | **0.29** | ****** |
|  |  | Black | **0.45** | ******* | **0.43** | ******* |
| Distress | Females | White | 0.04 | N.S. | 0.07 | N.S. |
|  |  | Black | **0.31** | ****** | **0.46** | ******* |
|  | Males | White | 0.06 | N.S. | 0.03 | N.S. |
|  |  | Black | 0.02 | N.S. | 0.09 | N.S. |

Note Sample includes 267 Males (46.8% White and 53.2% Black) and 462 Females (53.9% White and 46.1% Black). Results from unconstrained models; *p < .05. **p < .01. ***p < .001.
